# HLA-B*14:02-Restricted Env-Specific CD8^+^ T-Cell Activity Has Highly Potent Antiviral Efficacy Associated with Immune Control of HIV Infection

**DOI:** 10.1128/JVI.00544-17

**Published:** 2017-10-27

**Authors:** Ellen M. Leitman, Christian B. Willberg, Ming-Han Tsai, Huabiao Chen, Søren Buus, Fabian Chen, Lynn Riddell, David Haas, Jacques Fellay, James J. Goedert, Alicja Piechocka-Trocha, Bruce D. Walker, Jeffrey Martin, Steven Deeks, Steven M. Wolinsky, Jeremy Martinson, Maureen Martin, Ying Qi, Asier Sáez-Cirión, Otto O. Yang, Philippa C. Matthews, Mary Carrington, Philip J. R. Goulder

**Affiliations:** a Department of Paediatrics, University of Oxford, Oxford, United Kingdom; b Harvard Medical School, Boston, Massachusetts, USA; c Nuffield Department of Medicine, University of Oxford, Oxford, United Kingdom; d Ragon Institute of MGH, MIT and Harvard, Boston, Massachusetts, USA; e Vaccine and Immunotherapy Center, Massachusetts General Hospital and Harvard Medical School, Boston, Massachusetts, USA; f Laboratory of Experimental Immunology, Faculty of Health Sciences, University of Copenhagen, Copenhagen, Denmark; g Department of Sexual Health, Royal Berkshire Hospital, Reading, United Kingdom; h Integrated Sexual Health Services, Northamptonshire Healthcare NHS Trust, Northampton, United Kingdom; i Departments of Medicine, Pharmacology, Pathology, Microbiology, and Immunology, Vanderbilt University School of Medicine, Nashville, Tennessee, USA; j School of Life Sciences, Ecole Polytechnique Fédérale de Lausanne, Swiss Institute of Bioinformatics, Lausanne, Switzerland; k Infections and Immunoepidemiology Branch, Division of Cancer Epidemiology and Genetics, National Cancer Institute, National Institutes of Health, Bethesda, Maryland, USA; l HIV Pathogenesis Programme, The Doris Duke Medical Research Institute, University of KwaZulu-Natal, Durban, South Africa; m Department of Medicine, University of California San Francisco Department of Epidemiology and Biostatistics, University of California San Francisco, San Francisco, California, USA; n Department of Medicine, University of California, San Francisco, California, USA; o Division of Infectious Diseases, Northwestern University Feinberg School of Medicine, Chicago, Illinois, USA; p Department of Infectious Diseases and Microbiology, Graduate School of Public Health, University of Pittsburgh, Pittsburgh, Pennsylvania, USA; q Cancer and Inflammation Program, Leidos Biomedical Research, Frederick National Laboratory for Cancer Research, Frederick, Maryland, USA; r Institut Pasteur, Unité HIV, Inflammation et Persistance, Paris, France; s Department of Medicine, Geffen School of Medicine, University of California Los Angeles, Los Angeles, California, USA; t AIDS Healthcare Foundation, Los Angeles, California, USA; u Department of Infectious Diseases and Microbiology, Oxford University Hospitals NHS Foundation Trust, John Radcliffe Hospital, Oxford, United Kingdom; Ulm University Medical Center

**Keywords:** CD8^+^ T cells, HIV, HLA-B*14, immune control

## Abstract

Immune control of human immunodeficiency virus type 1 (HIV) infection is typically associated with effective Gag-specific CD8^+^ T-cell responses. We here focus on HLA-B*14, which protects against HIV disease progression, but the immunodominant HLA-B*14-restricted anti-HIV response is Env specific (ERYLKDQQL, HLA-B*14-EL9). A subdominant HLA-B*14-restricted response targets Gag (DRYFKTLRA, HLA-B*14-DA9). Using HLA-B*14/peptide-saporin-conjugated tetramers, we show that HLA-B*14-EL9 is substantially more potent at inhibiting viral replication than HLA-B*14-DA9. HLA-B*14-EL9 also has significantly higher functional avidity (*P* < 0.0001) and drives stronger selection pressure on the virus than HLA-B*14-DA9. However, these differences were HLA-B*14 subtype specific, applying only to HLA-B*14:02 and not to HLA-B*14:01. Furthermore, the HLA-B*14-associated protection against HIV disease progression is significantly greater for HLA-B*14:02 than for HLA-B*14:01, consistent with the superior antiviral efficacy of the HLA-B*14-EL9 response. Thus, although Gag-specific CD8^+^ T-cell responses may usually have greater anti-HIV efficacy, factors independent of protein specificity, including functional avidity of individual responses, are also critically important to immune control of HIV.

**IMPORTANCE** In HIV infection, although cytotoxic T lymphocytes (CTL) play a potentially critical role in eradication of viral reservoirs, the features that constitute an effective response remain poorly defined. We focus on HLA-B*14, unique among HLAs associated with control of HIV in that the dominant CTL response is Env specific, not Gag specific. We demonstrate that Env-specific HLA-B*14-restricted activity is substantially more efficacious than the subdominant HLA-B*14-restricted Gag response. Env immunodominance over Gag and strong Env-mediated selection pressure on HIV are observed only in subjects expressing HLA-B*14:02, and not HLA-B*14:01. This reflects the increased functional avidity of the Env response over Gag, substantially more marked for HLA-B*14:02. Finally, we show that HLA-B*14:02 is significantly more strongly associated with viremic control than HLA-B*14:01. These findings indicate that, although Gag-specific CTL may usually have greater anti-HIV efficacy than Env responses, factors independent of protein specificity, including functional avidity, may carry greater weight in mediating effective control of HIV.

## INTRODUCTION

Spontaneous durable control of HIV is observed in a rare subgroup (<1%) of infected individuals known as “elite controllers” (1). Nonprogressive HIV infection is associated with expression of certain HLA class I molecules (2, 3), such as HLA-B*57 and HLA-B*27 alleles (1, 4, 5). An important mechanism underlying the HLA associations with HIV disease outcome is related to the particular HIV-specific epitopes presented by different HLA class I molecules. In particular, “protective” HLA molecules typically present broad Gag-specific epitopes to CD8^+^ T cells, whereas disease-susceptible alleles such as HLA-B*35:01 and HLA-B*58:02 present Nef- and Env-specific epitopes, respectively, eliciting CD8^+^ T-cell responses that are typically associated with poor immune control of HIV (6–10).

Factors contributing to improved immune control in association with broad Gag and not Nef or Env responses include the sequence conservation especially of the capsid protein, because the cost to viral replicative capacity of Gag escape mutants is often significant (11–14). In contrast, Env escape mutants, for example, are typically tolerated by the virus without significant impact on viral replicative capacity (15). In addition, Gag capsid proteins are much more abundant than Env trimers in mature virions (1,000 to 1,500 per virion versus 10 to 20, respectively) (16), and Gag epitopes can be presented within 2 h of HIV gaining entry into the target cell, prior to *de novo* HIV protein synthesis (17). Hence, HIV-infected cells can be killed by Gag-specific CD8^+^ T cells before new virion production (17, 18). In contrast, Nef- and Env-specific CD8^+^ T-cell responses kill virus-infected target cells only after *de novo* synthesis of viral proteins (17–20) and therefore following Nef-mediated HLA class I downregulation (21, 22). Nonetheless, Gag-specific CD8^+^ T-cell responses are not equally efficacious (6, 23, 24), and there is evidence from the simian immunodeficiency virus (SIV)/macaque model that certain non-Gag epitopes, for example, within Nef and Vif, are important for immune control (25).

Furthermore, it is clear that several factors other than HIV protein specificity can play an important role in the efficacy of an epitope-specific response. These include functional avidity (26, 27), polyfunctionality (28), lytic granules (29), and proliferative capacity (30).

To investigate further the potential role of non-Gag-specific CD8^+^ T-cell responses in control of HIV infection, we focused here on HLA-B*14, where the dominant HIV-specific CD8^+^ T-cell response is in Env (31, 32). The association between HLA-B*14 and immune control of HIV has not been well studied to date (33), since most studies of elite controllers have focused on those expressing HLA-B*27 or -B*57 (26, 29, 30, 34–38). Although HLA-B*14 is not as strongly associated with HIV disease progression as HLA-B*27 or HLA-B*57, nonetheless, large studies have consistently shown a significant protective effect (3, 39–41). In addition to the dominant Env-specific CD8^+^ T-cell response, HLA-B*14-positive individuals also make a subdominant Gag-specific CD8^+^ T-cell response (42). We set out to investigate the role of these two specificities in HLA-B*14-mediated suppression of HIV and to understand the mechanisms underlying the observed differential antiviral activity among HLA-B*14-restricted CD8^+^ T-cell specificities.

## RESULTS

### Higher antiviral potency of B*14:02-Env-EL9 than of -Gag-DA9 CD8^+^ T-cell response.

**FIG 1:**
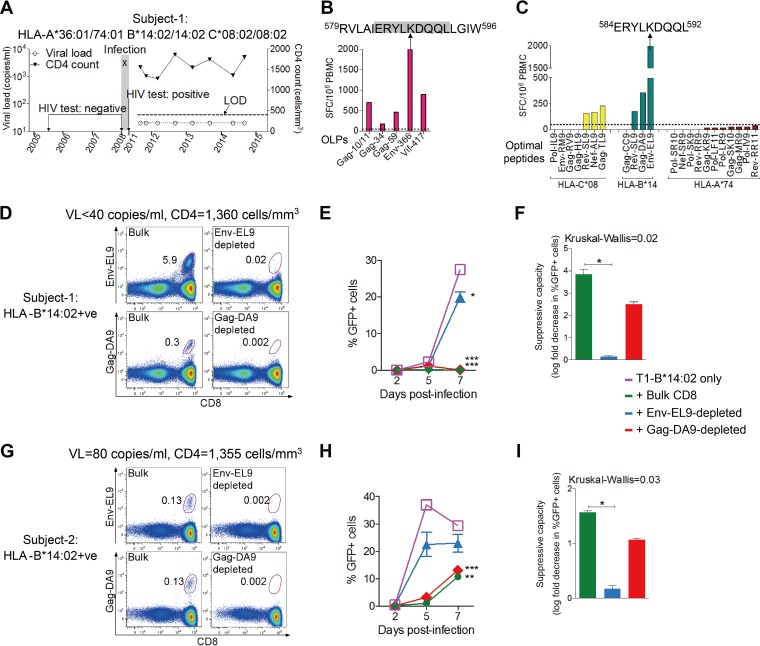
Higher antiviral potency of B*14:02-EL9 than of -DA9 CD8 T-cell response. (A) HIV-related clinical profile of subject 1; gray area shows time period during which infection occurred. All viral load measurements were undetectable (<40 copies/ml) and are shown below the limit of detection (LOD) of 40 copies/ml for convenience. (B) CD8^+^ T-cell IFN-γ ELISPOT responses to overlapping peptides (OLPs) spanning the entire HIV proteome in subject 1. The dotted line shows the cutoff magnitude (50 SFC/10^6^ PBMC). (C) CD8^+^ T-cell IFN-γ ELISPOT responses to epitopes restricted by HLA class I alleles expressed by subject 1. HLA-A*36:01-restricted responses are not shown as these are not defined. The dotted line shows the cutoff magnitude (50 SFC/10^6^ PBMC). (D to F) Data for subject 1. (G to I) Data for subject 2. (D and G) Tetramer stainings confirming HLA-B*14-02-Env-EL9 (top panels) and HLA-B*14:02-Gag-DA9 (bottom panels) CD8^+^ T-cell responses in bulk (left panels) and tetramer-depleted (right panels) cultures. Gated on live CD3^+^ CD4^−^ lymphocytes around CD8^+^ tetramer^+^ cells; numbers indicate percentage of CD8^+^ cells. (E and H) Viral replication (percent GFP^+^ cells) time course in infected T1-HLA-B*14:02-positive target cells with or without effector CD8^+^ T cells. Results were compared to T1-HLA-B*14:02 target cells only at the peak of viral replication using paired *t* tests. (F and I) Suppressive capacity of bulk Env-EL9-depleted or Gag-DA9-depleted effector cells calculated as described in Materials and Methods. Significance was determined by Kruskal-Wallis test with Dunn's multiple-comparison test. (E, F, H, and I) Error bars represent standard errors of the means. *, *P* < 0.05; **, *P* < 0.01; ***, *P* < 0.001. Only significant differences are shown. The color key applies to panels E, F, H, and I.

The starting point for this study was an elite controller subject, subject 1, who first tested HIV positive in the United Kingdom in 2011, having previously had two negative tests in 2005 and 2008 (Fig. 1A). Since the positive HIV test, subject 1 maintained an undetectable viral load (VL; <40 copies/ml) and healthy and stable CD4^+^ T-cell counts (median, 1,555 cells/mm^3^; interquartile range [IQR], 1,345 to 1,788). Viral sequencing revealed that she was infected with subtype B virus. HLA genotyping showed that she was HLA-B*14:02/HLA-C*08:02 homozygous and also expressed another HLA molecule, HLA-A*74:01, associated with slow disease progression (43).

To investigate the role that HLA-B*14:02-restricted CD8^+^ T-cell responses might play in immune control of HIV, we first screened peripheral blood mononuclear cells (PBMC) in this individual for HIV-specific gamma interferon (IFN-γ) enzyme-linked immunosorbent spot (ELISPOT) assay responses using overlapping peptides spanning the entire HIV proteome (39) together with previously defined HIV-specific epitopes (44). The HLA-B*14:02-restricted responses dominated overall, the highest-magnitude responses being those to the HLA-B*14:02-restricted Env-EL9 (^584^ERYLKDQQL^592^) (31) and its corresponding overlapping peptide Env-366 (^579^RVLAIERYLKDQQLLGIW^596^) (Fig. 1B and C). The next highest optimal peptide response was toward the HLA-B*14:02-Gag-DA9 epitope (^298^DRFYKTLRA^306^) (42).

To test the hypothesis that suppression of HIV in this patient was mediated principally by HLA-B*14:02-restricted CD8^+^ T-cell activity, we next compared the antiviral potencies of Env-EL9- and Gag-DA9-specific CD8^+^ T cells. From this same elite controller (subject 1), bulk CD8^+^ T cells were first expanded with the bispecific CD3.4 antibody (45–47) and then depleted of selected CD8^+^ T-cell specificities using cytotoxic saporin (SAP)-conjugated tetramers (tet-SAP; see Materials and Methods) (Fig. 1D). The ability of the bulk or depleted cytotoxic T lymphocytes (CTL) to inhibit viral replication *in vitro* was then evaluated using T1 cells expressing HLA-B*14:02 as CD4^+^ T-cell targets and the B clade NL4-3 as the test strain of HIV (Fig. 1E and F). Removal of the Env-EL9 specificity substantially reduced the HIV-suppressive capacity of the expanded CD8^+^ T cells (22% of target cells infected versus 0.001% [Fig. 1E]), and suppressive capacity was reduced by 26-fold (bulk CD8, 3.85 log_10_, versus Env-EL9-depleted CD8, 0.15 log_10_) (Kruskal-Wallis, *P* = 0.02) (Fig. 1F). In contrast, depletion of Gag-DA9-depleted cells made little impact. This result suggests that the presence of Env-EL9 specificity represents the majority of CD8^+^ T-cell-mediated control of viral suppression in subject 1 and that the Gag-DA9 specificity does not contribute significantly.

A potential caveat of this finding in this study subject is the unequal frequency of Env- and Gag-specific CD8^+^ T cells, with Env-specific cells being nearly 20-fold more frequent than Gag-specific cells (Fig. 1D). To address this matter, we adopted two approaches. First, we repeated targeted depletion experiments using cells from another chronically B-clade-infected HLA-B*14:02-positive controller (subject 2, viral load [VL], 80 copies/ml; CD4, 1,355 cells/mm^3^), who had equal frequencies of Env-EL9- and Gag-DA9-specific CD8^+^ T cells (Fig. 1G, left panels). As with subject 1, elimination of the Env-EL9 specificity resulted in preservation of 25% of infected target cells at the peak of viral replication compared to only 1% in bulk CD8^+^ T-cell coculture (Fig. 1H), representing a 16-fold reduction (1.6 log_10_ versus 0.1 log_10_) of suppressive capacity of the Env-EL9-depleted CTL (Kruskal-Wallis, *P* = 0.03) (Fig. 1I). In contrast, Gag-DA9 depletion had only a minor effect, thus supporting the notion that Env-EL9-specific CTL mediate the major antiviral efficacy of the two HLA-B*14:02-restricted responses.

**FIG 2:**
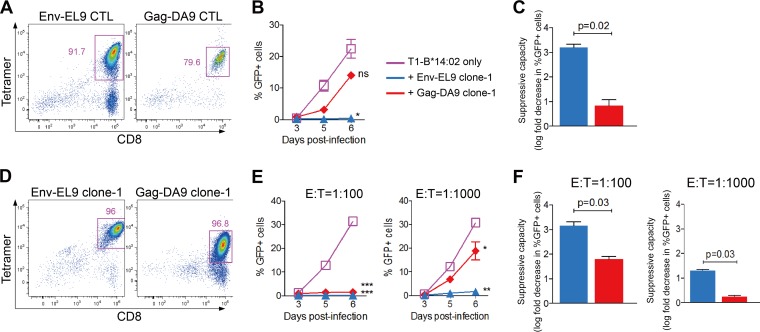
Suppressive capacity of epitope-specific Env and Gag CTL lines and clones. (A to C) Results for CTL lines, generated by peptide stimulation of epitope-specific cells from PBMC, with sorting and further expansion before testing in inhibition assays. (D to F) Examples of clones, generated by single-cell sorting epitope-specific cells and growing them out in culture before testing their antiviral capacity. (A and D) Confirmatory tetramer stainings of epitope-specific CTL lines (A) and clones (D). Gated on live CD3^+^ CD4^−^ cells around CD8^+^ tetramer^+^ cells; numbers indicate percent tetramer^+^ cells (of CD3^+^ CD4^−^ cells). (B and E) Viral replication in infected T1-HLA-B*14:02-positive target cells with or without effector cells. Results were compared to T1-HLA-B*14:02 target cells only at the peak of viral replication using paired *t* tests. *, *P* < 0.05; **, *P* < 0.01; ***, *P* < 0.001, ns, not significant (*P* > 0.05). (C and F) Suppressive capacity of effector cells. Significance was determined by Mann-Whitney U test. (B, C, E, and F) Error bars represent standard errors of the means. The color key in panel B applies also to panels C, E, and F. E:T, effector/target ratio.

In the second approach, we tested the antiviral efficacy of Env-EL9- and Gag-DA9-specific CD8^+^ T cells directly by generating epitope-specific CTL lines and clones. In the experiments using peptide-specific lines (Fig. 2A to C), once again Env-EL9-specific CTL were significantly more potent at suppressing viral replication at the same effector-to-target ratio of 1:100 (*P* = 0.02) (Fig. 2B and C). Similarly, Env-EL9-specific CTL clones were more potent inhibitors of viral replication than were Gag-DA9 clones. This was particularly evident at the lower effector-to-target ratios (1:1,000) (Fig. 2D to F). Taken together, these experiments suggest that Env-EL9-specific CD8^+^ T cells are more efficacious at suppressing HIV replication than Gag-DA9-specific cells.

### Higher functional avidity, antigen recognition, and magnitude of EL9 than of DA9.

**FIG 3:**
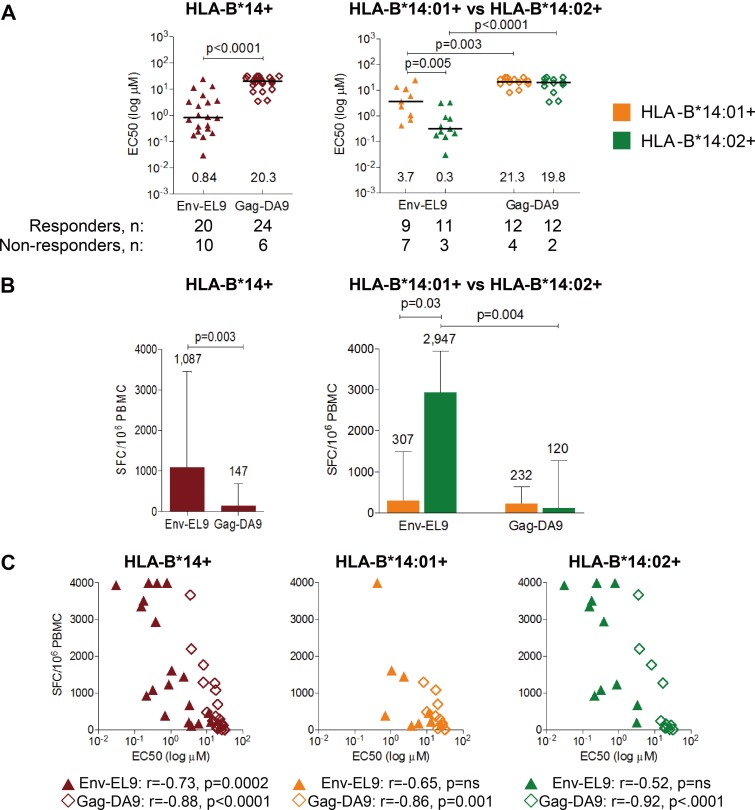
Higher functional avidity and magnitude of EL9- than of DA9-specific response. (A) Functional avidity (EC_50_) of Env-EL9 versus Gag-DA9 CD8^+^ T-cell responses in all HLA-B*14-positive subjects (*n* = 30, left panel) or separately in HLA-B*14:01-expressing (*n* = 16) or HLA-B*14:02-expressing (*n* = 14) subjects (right panel). Lines and numbers indicate median values. Significance was determined by Mann-Whitney U tests. (B) Magnitude of Env-EL9 or Gag-DA9 responses determined by IFN-γ ELISPOT assay in all HLA-B*14-positive subjects (*n* = 30; left panel) or in HLA-B*14:01-expressing (*n* = 16) and HLA-B*14:02-expressing (*n* = 14) subjects (right panel). Numbers above the bar graphs indicate median values; error bars show interquartile ranges. Significance was determined by Mann-Whitney U tests. (C) Correlation between response magnitude and functional avidity in HLA-B*14-positive (left), HLA-B*14:01-positive (middle), and HLA-B*14:02-positive subjects. *r* and *P* values were obtained by Spearman correlation. The color key for all panels is shown in panel A.

In order to further investigate this observed antiviral superiority of Env-EL9-specific cells over Gag-DA9-specific cells, we next examined the functional avidity of the two specificities (determined by the peptide concentration required for 50% maximal recognition [EC_50_]), the response magnitude, and the frequency of epitope recognition in a larger number of HLA-B*14-positive subjects (*n* = 30). Among all HLA-B*14-positive subjects, functional avidity, or antigen sensitivity (EC_50_), of the Env-EL9 response was >24-fold higher than that of the Gag-DA9 response (median, 0.84 versus 20.3 μM; *P* < 0.0001) (Fig. 3A, left panel). This difference was significant among both HLA-B*14:01-positive (median, 3.7 versus 21.3 μM; *P* = 0.003) and HLA-B*14:02-positive (median, 0.3 versus 19.8 μM; *P* < 0.0001) (Fig. 3A, right panel) subjects. However, Env-EL9 functional avidity was 12-fold higher in HLA-B*14:02-positive subjects than in HLA-B*14:01-positive subjects (*P* = 0.005) (Fig. 3A, right panel).

The magnitude of the Env-EL9 response was also >9-fold higher than that of the Gag-DA9 response among all HLA-B*14-expressing subjects among responders (*P* = 0.003) (Fig. 3B, left panel). This difference was significant only among HLA-B*14:02-positive subjects (Fig. 3B, right panel). Additionally, HLA-B*14:02-positive subjects had a significantly higher magnitude of the Env-EL9 response than the HLA-B*14:01-positive subjects (*P* = 0.03). Interestingly, the magnitude of both Env-EL9 and Gag-DA9 responses was negatively correlated with EC_50_ (i.e., positively with functional avidity) (Env-EL9, *r* = −0.73, *P* = 0.0002; Gag-DA9, *r* = −0.88, *P* < 0.0001) (Fig. 3C), indicating that cells with higher functional avidity mounted a response of greater magnitude.

Together, these data demonstrate that the greater antiviral potency of the HLA-B*14-Env-EL9-specific response observed above is also associated with higher functional avidity and response magnitude than those of the Gag-DA9-specific response.

### Differential Env-EL9 and Gag-DA9 selection pressure in B*14:01 versus B*14:02.

**FIG 4:**
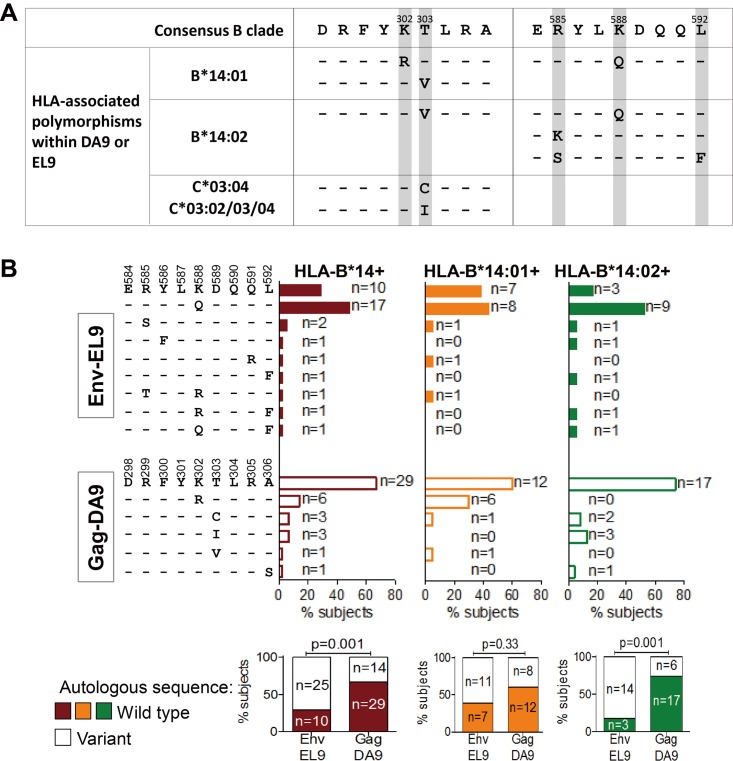
Differential Env-EL9 and Gag-DA9 selection pressure in B*14:01 versus B*14:02. (A) Consensus sequences of Env-EL9 and Gag-DA9 epitopes and polymorphisms associated with HLA-B*14 subtypes; overlapping polymorphisms associated with non-HLA-B*14 alleles are also shown. Data compiled from previously published large cohort studies (48–50). (B) Frequency of Env-EL9 and Gag-DA9 wild-type and variant sequences in the studied HLA-B*14-positive subjects. Graphs at the bottom compare frequencies of subjects with autologous wild-type (filled bars) or mutated (empty bars) sequence of Env-EL9 versus Gag-DA9 epitopes. Significance was determined by Fisher's exact tests. (A and B) Residues identical to the wild type are presented as dashes; nonidentical residues are specified.

To further understand the differences between Env-EL9- and Gag-DA9-specific CD8^+^ T-cell functions, we next investigated what selection pressure is imposed on the virus by these two responses (Fig. 4). Consistent with previously published data describing HLA-associated polymorphisms from analysis of 3,754 HLA-typed treatment-naive persons (48, 49) within the Env-EL9 epitope (Fig. 4A), K588Q is strongly selected among both HLA-B*14:01- and HLA-B*14:02-positive persons, and variants at Env-588 and at other residues within Env-EL9 are observed more commonly in HLA-B*14:02-positive subjects (although here these differences between HLA-B*14:01 and HLA-B*14:02 did not reach statistical significance) (Fig. 4B). These Env-EL9 sequence data indicate stronger selection pressure imposed on the virus by the HLA-B*14:02-EL9 response than by the HLA-B*14:01-restricted EL9 response.

To put these Env-EL9 variants arising in HLA-B*14-expressing individuals into the context of variation within this epitope overall, the frequency of Env-EL9 variants in B- and C-clade infection in all subjects (https://www.hiv.lanl.gov) is 50% and 34%, respectively, the most frequent variants being K558R (29% and 9% in B- and C-clade sequences, respectively) and K588Q (8% and 16%, respectively). Thus, K588R is relatively common and, as shown in Fig. 4A, is not an HLA-B*14:01 or HLA-B*14:02 footprint and indeed is not selected without accompanying variants in any of the subjects studied here, whereas K588Q is selected in 17/35 (49%) HLA-B*14-positive subjects studied here and in 17/25 (68%) of those whose autologous virus encoded Env-EL9 variants.

For the Gag-DA9 epitope, as shown in previous large cohort studies (48–50), the most frequent K302R variant is selected only in HLA-B*14:01-positive subjects (*P* = 0.01). Thus, there was significantly more variation in HLA-B*14:02-positive subjects in the Env-EL9 epitope than in Gag-DA9 (*P* = 0.001) (Fig. 4B), consistent with this being the dominant response among HLA-B*14:02-positive subjects. Among B*14:01-positive subjects, the Env-EL9 epitope was not targeted significantly more than Gag-DA9, and correspondingly, there was no significant difference in the selection of variants within Env-EL9 and Gag-DA9 in these subjects.

### Selection of K588Q and not K588R is an escape variant in HLA-B*14-positive subjects.

The Env-EL9 sequence data shown above confirm previous studies showing that K588Q at position 5 (P5) in the epitope and binding in the D pocket of the HLA-B*14 peptide-binding groove (51) is an HLA-B*14 footprint but K588R is not (48). This is surprising, given the relatively frequent occurrence of Arg/Lys substitutions as a mechanism by which HIV can escape recognition by other CTL specificities. Indeed, the HLA-B*14:01-associated escape variant within Gag-DA9 is a case in point, K302R being the substitution characteristically selected at P5 in the epitope.

**FIG 5:**
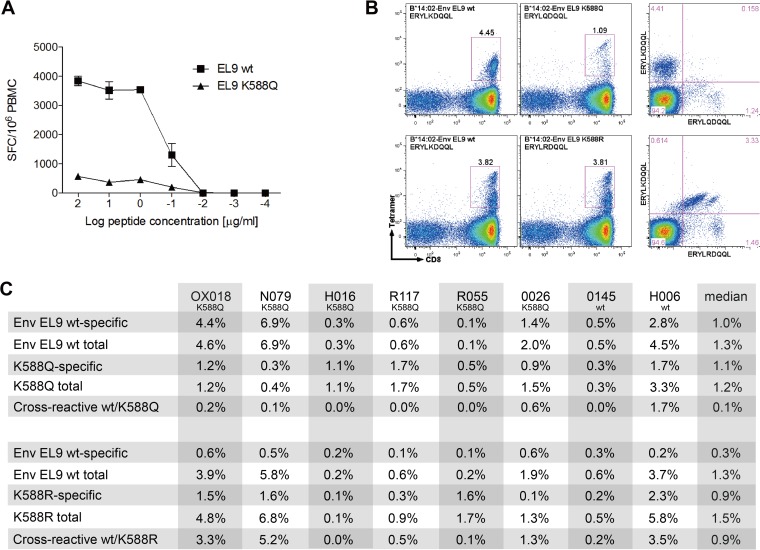
K588Q and not K588R is an escape variant in HLA-B*14-positive subjects. (A) Representative example of responses to EL9 wild-type versus EL9 K588Q variant peptides at different peptide concentrations by IFN-γ ELISPOT assay. The example shown is subject OX018, who has an autologous EL9 K588Q variant. (B) Cross-recognition of EL9 wild type and EL9 K588Q (top panels) versus EL9 wild type and EL9 K588R (bottom panels). The example shown is subject OX018, who has an autologous EL9 K588Q variant. (C) Cross-reactivity data of wild-type EL9-specific cells with K588Q and K588R variants for 8 H:A-B*14-positive subjects determined by tetramer staining.

In order to address this question, the ability of Env-EL9-specific CD8^+^ T cells to cross-react with the K588Q and with the K588R variants was analyzed in 8 HLA-B*14-positive subjects for whom samples were available. In 6 of 8 subjects, the autologous variant was K588Q, and in the remaining two, autologous virus encoded wild-type (wt) Env-EL9. The pattern of cross-recognition observed was quite distinct for the two variants K588Q and K558R. For K588Q, in all 8 subjects, the frequency of cross-reactive cells, double stained by EL9-wt and EL9-K558Q tetramers, was substantially lower than the frequency of EL9-wt-specific CD8^+^ T cells (Fig. 5). In all cases, including the 6 in whom the K588Q variant had been selected, EL9-wt-specific responses were readily detectable and in 5 of 8 cases were greater in magnitude than the EL9-K588Q-specific response. In contrast, in most of these subjects (6 of 8), the frequency of CTL cross-reactive for EL9-wt and K588R was higher than that of EL9-wt-specific CTL. Indeed, in 6 of these 8 subjects the K588R variant-specific response was higher than the EL9-wt-specific response.

These results would explain why K588R is not selected as an escape mutant, since the EL9-wt-specific response typically cross-reacts strongly with the K588R variant. In contrast, the K588Q variant is not cross-recognized, and therefore, this mutant would carry a selective advantage for the virus.

### Impact of Gag-DA9 and Env-EL9 escape mutants on viral fitness and HIV outcome.

**FIG 6:**
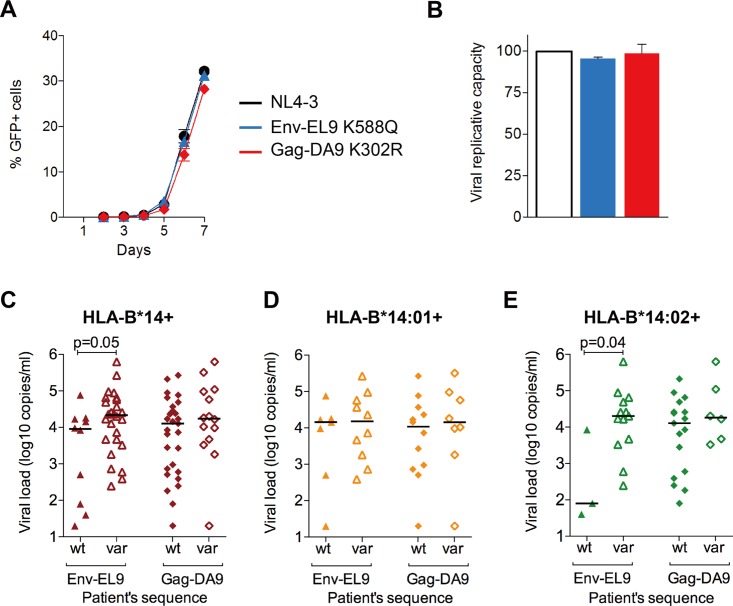
Impact of Gag-DA9 and Env-EL9 escape mutants on viral fitness and HIV infection outcome. (A) Replication kinetics of NL4-3 containing wild-type p24 and Env compared to the C-clade version of Gag-DA9 epitope and three other HLA-B*14-associated Gag and Env mutants. Infectivity is expressed as percent GFP-positive GXR reporter cells over 7 days after infection. Error bars represent standard errors of the means. (B) Viral replication capacity of the viruses in panel A. Significance was determined by ANOVA with Dunnett's multiple-comparison test comparing NL4-3 with the other viruses. Error bars represent standard errors of the means. *, *P* < 0.05; **, *P* < 0.01; ***, *P* < 0.001; ns, not significant (*P* > 0.05). The color code is as in panel A. (C to E) Viral loads in HLA-B*14-positive (C), HLA-B*14:01-positive (D), and HLA-B*14:02-positive (E) subjects with either wild-type or mutated autologous sequences in Env-EL9 and Gag-DA9 epitopes. *x* axes indicate patients' autologous epitope sequences. Only significant *P* values (*P* > 0.05), obtained by the Mann-Whitney U test, are shown. Horizontal bars indicate medians. wt, wild type; var, variant sequence.

Previous studies have indicated that the most effective CTL responses are those capable of driving the selection of escape mutants that significantly reduce viral replicative capacity (11–14). These are more likely in p24 Gag, which is highly conserved, than in Env, which is highly variable. We next, therefore, investigated the impact on viral replicative capacity of the most common escape mutants in p24 Gag-DA9 and Env-EL9 (Fig. 6A and B). In contrast to the general observation of the high cost of p24 Gag mutants, the K302R mutant had little impact on viral replicative capacity (VRC), consistent with previous studies of this variant (52). The Env-EL9 mutant marginally but not significantly decreased VRC. Thus, in this particular case, the fitness cost resulting from the selection of escape mutants within the capsid protein appears to have little impact on viral replicative capacity.

HLA-B*14-positive subjects who had wild-type Env-EL9 had lower viral loads (median of 9,068 versus 21,546 copies/ml, *P* = 0.05) (Fig. 6C) and higher CD4^+^ T-cell counts (median of 606 versus 455 cells/mm^3^, *P* = 0.005). Again, this difference was exclusive to HLA-B*14:02-positive subjects, among whom individuals with wild-type Env-EL9 had lower VLs than did those with a variant (median of 80 versus 20,003 copies/ml, *P* = 0.04) (Fig. 6D and E) and a trend toward higher CD4^+^ T-cell counts (median of 1,340 versus 410 cells/mm^3^, *P* = 0.2), although there were only three subjects with wild-type sequence.

Taken together, these results suggest that the Env-EL9 response is highly effective and contributes to successful immune control of HIV, whereas Gag-DA9 is not.

### Stronger association of HLA-B*14:02 than HLA-B*14:01 with HIV immune control.

On the basis of the findings above, if the B*14-Env-EL9 response makes a significant contribution to immune control of HIV, one would predict greater protection against HIV disease progression in association with HLA-B*14:02 than with HLA-B*14:01. To test this hypothesis, we first compared the frequency of HLA-B*14:01 with that of HLA-B*14:02 in viremic controllers (viral load of <2,000 copies/ml) versus noncontrollers (viral load of >10,000 copies/ml) derived from several previously well-described cohorts (see Materials and Methods). Although there was a trend toward HLA-B*14:02 being more protective than HLA-B*14:01 among HLA-B*14-positive Caucasians (self-identified as “white”; *n* = 285) and HLA-B*14-positive African Americans (self-identified as “black”; *n* = 104), in neither group was the difference statistically significant (Table 1). We then extended the analysis to all controllers and noncontrollers in our cohorts (whites, *n* = 3,259; blacks, *n* = 1,745) by performing a regression analysis with stepwise selection that included all HLA-B alleles having a frequency equal to or greater than that of HLA-B*14:01 (HLA-B*14:01 frequency in whites, 2.42%; in blacks, 1.60%) (Table 2). HLA-B*14:02 showed a significant independent protective effect in both whites (odds ratio [OR], 0.44; *P* = 2e−7 in whites) and blacks (OR, 0.54; *P* = 2e−2 in blacks), but HLA-B*14:01 did not in either group. These results are consistent with previous data where HLA-B*14:02 was shown to confer protection in a logistic regression model that included all HLA class I alleles with a phenotypic frequency of >2% (33) and with our immunological findings.

**TABLE 1:** Frequency of HLA-B*14:01 versus HLA-B*14:02 among viremic controllers and noncontrollers^*a*^

Patient group, allele, and adjustment	No. of controllers	No. of noncontrollers	B*14:01 vs B*14:02	
			OR (95% CI)	*P* value
HLA-B*14^+^ (*n* = 285) from 3,259 whites				
B*14:01	23	54		
B*14:02	78	130		
Not adjusted			1.41 (0.80–2.47)	0.23
Adjusted for B*27, B*57			1.54 (0.86–2.77)	0.15
HLA-B*14^+^ (*n* = 104) from 1,745 blacks				
B*14:01	7	21		
B*14:02	30	46		
Not adjusted			1.96 (0.74–5.17)	0.18
Adjusted for B*57, B*81			1.52 (0.56–4.13)	0.41

a ART-naive chronically HIV-infected subjects were categorized as viremic controllers (viral loads of <2,000 copies/ml plasma) or noncontrollers (viral loads of >10,000 copies/ml plasma). Logistic regression was used to compute significance (*P* values), OR, and 95% CI for the differences in frequency of HLA-B*14 subtypes in controllers versus noncontrollers, with adjustment by HLA-B*57/27 expression.

**TABLE 2:** HLA-B*14:02 is significantly enriched among viremic controllers^*a*^

Patient group and allele being compared with others	OR	95% CI	*P* value
Whites (*n* = 3,259)			
B*57:01	0.25	0.20–0.32	2e−31
B*27:05	0.34	0.26–0.45	2e−15
B*52:01	0.40	0.25–0.63	7e−5
B*14:02	0.44	0.32–0.60	2e−7
B*13:02	0.47	0.33–0.67	3e−5
B*40:02	0.48	0.31–0.75	1e−3
B*08:01	1.66	1.28–2.13	1e−4
B*38:01	1.66	1.04–2.66	3e−2
B*40:01	1.76	1.24–2.50	2e−3
B*07:02	2.04	1.60–2.60	8e−9
Blacks (*n* = 1,745)			
B*57:03	0.15	0.11–0.21	2e−29
B*81:01	0.20	0.12–0.33	1e−10
B*39:10	0.22	0.11–0.45	2e−5
B*57:01	0.41	0.19–0.91	3e−2
B*27:05	0.44	0.21–0.91	3e−2
B*14:02	0.54	0.32–0.90	2e−2
B*07:02	1.45	1.01–2.09	4e−2
B*53:01	1.51	1.12–2.03	7e−3
B*35:01	1.92	1.29–2.86	1e−3
B*15:10	2.27	1.33–3.89	3e−3
B*58:02	2.63	1.51–4.59	6e−4
B*45:01	4.10	2.36–7.13	6e−7

a Presence or absence of individual HLA-B alleles that have a frequency equal to or greater than that of HLA-B*14:01 was tested by logistic regression with stepwise selection. B*14:02 is highlighted in bold.

## DISCUSSION

This study investigated HIV control mediated by HLA-B*14. We showed that the HLA-B*14-restricted Env-EL9-specific CD8^+^ T-cell response was more efficacious against HIV than the Gag-DA9-specific response. In association with this, the Env-specific response had significantly greater function avidity, was more frequently targeted, and was of higher magnitude than the Gag-specific response. We demonstrated that the functional avidity for Env-EL9 was significantly higher for responses restricted by HLA-B*14:02 than for responses restricted by HLA-B*14:01. Finally, we showed a significantly stronger association between HLA-B*14:02 and protection against HIV disease progression than with the protection mediated by HLA-B*14:01.

Higher anti-HIV efficacy of Env- than of Gag-specific CD8^+^ T-cell responses is unusual and, in fact, unreported until the current study. Numerous studies have suggested that Gag-specific CD8^+^ T-cell responses are associated with better disease outcome in HIV infection, are more efficacious in control of HIV than other specificities, and are often dominant responses in elite controllers (6, 10, 18, 53–64). However, our result is consistent with studies showing that non-Gag responses can also mediate viral control. In the macaque model of elite control, Env-, Nef-, and Vif-specific CD8^+^ T-cell responses have been shown to be dominant and efficacious in viral clearance (25, 65, 66). In HIV infection, a dominant HLA-B*57-restricted Nef response was present in an elite controller, although the antiviral efficacy of that response has not been evaluated (67). Similarly, HLA-B*27:02, an allele associated with better protection than HLA-B*27:05 (P. Goulder, unpublished data), restricts the dominant CD8^+^ T-cell response in Nef, although as described above its efficacy remains to be determined. Furthermore, effective elimination of infected cells by Env-specific CD8^+^ T cells, including HLA-B*14-Env-EL9-restricted cells, has been reported in HIV infection (68, 69). Our finding of the superior Env-specific antiviral efficacy is also consistent with a recent study of HLA-B*57/27-negative HIV elite controllers indicating that potent cytotoxic capacity (measured by granzyme B expression and infected cell elimination) of HIV-specific CD8^+^ T cells rather than the identity of the targeted epitope is the determining factor in mediating successful control of infection (70).

The data presented here are consistent with previous studies suggesting that an important factor contributing to antiviral efficacy of CD8^+^ T-cell responses is high functional avidity (26, 27, 71, 72). Functional avidity of the Env-EL9 response was >24-fold higher than that of the Gag-DA9-specific response and correlated strongly with response magnitude; the Env-specific response was also more frequently targeted than the Gag-specific response (Fig. 3). Thus, HLA-B*14-Env-EL9 CD8^+^ T cells with higher antigen sensitivity than HLA-B*14-Gag-DA9 cells would be expected to be more efficacious in controlling viral replication.

These qualitative differences between Env- and Gag-specific CD8^+^ T cells were significant only among HLA-B*14:02-positive individuals (Fig. 3). In the case of functional avidity, the difference between the Env- and Gag-specific responses was significant even among HLA-B*14:01-positive individuals, although less markedly so. However, the avidity of the Env-EL9 response was still 12-fold higher in the HLA-B*14:02-positive subjects than in the HLA-B*14:01-positive subjects. These observations suggest that despite restriction of the same epitopes by these two closely related HLA-B*14 molecules, HIV-specific HLA-B*14:02-restricted CD8^+^ T cells are qualitatively different from HIV-specific HLA-B*14:01-restricted CD8^+^ T cells, and the difference is primarily determined by the superior function of HLA-B*14:02-restricted Env-EL9-specific CD8^+^ T cells.

It has previously been proposed that an important mechanism by which HLA class I molecules influence rates of HIV disease progression is related to the specificity of the particular HIV epitopes that are presented (8). As described above, factors other than specificity are important. However, the demonstration here of the HLA-B*14-EL9 response as both immunodominant among HLA-B*14-positive subjects and efficacious in suppressing HIV is consistent with previous observations of HLA-B*14 being associated with protection against rapid HIV disease progression (3, 39, 41). The substantially higher functional avidity of this Env-EL9 response among HLA-B*14:02- than HLA-B*14:01-positive subjects is also consistent with the findings here that HLA-B*14:02 is significantly more protective against HIV disease progression than HLA-B*14:01.

These studies have focused on the two principal HLA-B*14-restricted HIV-specific responses. We have not considered other HLA-B*14-restricted HIV-specific responses since these are the only 2 that drive selection pressure on the virus (48). The labor-intensive nature of the work and the consequently small number of subjects studied here limit our concluding definitively that in all cases HLA-B*14-restricted Env-EL9-specific CD8^+^ T cells inhibit HIV replication more effectively than HLA-B*14-restricted Gag-DA9-specific responses. In addition, sample availability precluded our comparing the capacities of HLA-B*14:01-restricted CTL clones and HLA-B*14:02-restricted CTL clones to inhibit viral replication.

Structurally, HLA-B*14:01 and HLA-B*14:02 differ only at position 11 (P11), with a serine and an alanine, respectively (73). However, P11 is unlikely to explain the difference in HIV control between the two HLA molecules, because of its “buried” location in the α1 domain of HLA near the C pocket (74, 75), where it does not contribute to interactions between HLA domains or with a peptide or T-cell receptor (TCR), is not solvent accessible, and is of low variability (76, 77). The peptide-binding motif for HLA-B*14:02 has previously been determined (51). Studies identifying HLA-B*14-restricted epitopes reported only 2-digit HLA, thus not showing an HLA-B*14:01 motif explicitly (31, 42). However, due to the lack of significant structural differences the peptide-binding motif is likely to be the same for the two molecules.

On the other hand, the buried P11 may alter the confirmation of the nearby α1 residues or affect upstream peptide processing (73). Of note, HLA-B*14:02 and HLA-B*14:03 differ by a single amino acid in the HLA sequence at P156 but share only ∼30% of their peptides (78). This single difference at P156, essential in D and E peptide-binding pockets (76), may play a role in the differential association of HLA-B*14:02 and HLA-B*14:03 with ankylosing spondylitis (78, 79). Previous HIV-specific studies have also shown that one amino acid difference between HLA subtypes, such as HLA-B*35:01 and HLA-B*35:03 (80), or HLA-B*35:01 and HLA-B*35:08 (81), HLA-B*42:01 and HLA-B*42:02 (24), and HLA-B*57:02 and HLA-B*57:03 (82), is sufficient to have a significant impact on disease outcome.

Another distinguishing feature between HLA-B*14:01 and HLA-B*14:02 alleles appears to be in the selection pressure that they exert on the virus. First, the Gag-DA9 K302R mutation was found exclusively in HLA-B*14:01-positive subjects, consistent with previous large cohort studies involving >3,500 study subjects (48, 49). This again would point to the lack of antiviral efficacy of HLA-B*14:02-Gag-specific CD8^+^ T cells. At the same time, HLA-B*14:02 appeared to have selected the Env-EL9 K588Q mutation, and there is a hint that this selection is associated with higher viremia and lower CD4^+^ T-cell counts (Fig. 6C to E). Curiously, however, neither of these mutations had a significant impact on viral replication, although Env-EL9-K588Q tended to have a slightly lower replicative capacity than the wild type, while the opposite was true for Gag-DA9-K302R (Fig. 6). This is particularly interesting in the case of the Gag epitope. This epitope (Gag 298 to 306) overlaps with the highly conserved major homology region (Gag 285 to 304) in the C terminus of HIV p24 capsid, which is essential for virion assembly and stability (83, 84), and previous reports showed rapid reversion of K302R in the absence of HLA-B*14:01 (50, 85), implying that K302R inflicts a significant cost to viral replicative capacity. However, the apparent lack of impact of this mutation on viral replication was also recently reported by another group (52).

It is perhaps surprising that previous large studies investigating the relationship between HLA class I type and HIV disease progression did not identify the difference between HLA-B*14:02 and HLA-B*14:01 in terms of the protective effect conferred. In some earlier studies, 2-digit HLA typing was employed (41), which prevented these analyses being undertaken. Also, HLA-B*14 class I subtypes are not especially prevalent, especially in African populations (the phenotypic frequency of HLA-B*14 is 6.0% versus 8.7% in the present study [Tables 1 and 2]), and hence, large study numbers are needed to achieve adequate statistical power. The current analysis involved 3,259 whites and 1,745 blacks, and even with these numbers, the protection afforded by HLA-B*14:02 in the blacks was evident only at a *P* value of 0.02.

In conclusion, these studies indicate that, although Gag-specific CD8^+^ T-cell responses may usually have greater antiviral efficacy against HIV for the several reasons described above, influences such as functional avidity of individual responses are also critically important factors that may override protein specificity in contributing to immune control of HIV infection. This finding is relevant to the development of vaccines designed to generate effective antiviral CD8^+^ T-cell responses.

## MATERIALS AND METHODS

### Study subjects.

Adult chronically HIV-infected antiretroviral therapy (ART)-naive subjects studied here were enrolled in the following cohorts: (i) Thames Valley cohort, United Kingdom (*n* = 30) (18); (ii) Gateway cohort, Durban, South Africa (*n* = 17) (86); and (iii) the SCOPE (Study of the Consequences of Protease Inhibitor Era) cohort, San Francisco, CA, USA (*n* = 2) (87). Subjects from all cohorts provided written informed consent, and the study was approved by the institutional boards of the University of Oxford, the University of KwaZulu-Natal, and the University of California, San Francisco. HLA typing was performed using a locus-specific PCR amplification strategy and a heterozygous DNA sequencing methodology for the HLA class I exon 2 and 3 amplicons. HIV plasma viral load measurements were done using the Roche Amplicor version 1.5 assay with Cobas Amplicor (Thames Valley, Gateway, and SCOPE cohorts) or using the Abbott RealTime HIV assay (SCOPE cohort). CD4^+^ T-cell counts were enumerated by flow cytometry using standard clinical protocol. The median viral load of these study subjects was 9,700 copies/ml (IQR, 555 to 31,500); the median CD4^+^ T-cell count was 527 cells/mm^3^ (IQR, 420 to 711).

To analyze the associations between the expression of HLA-B*14:01 and HLA-B*14:02 and immune control of HIV, viral load and HLA data of the ART-naive chronically HIV-infected subjects (*n* = 5,004; Caucasians, *n* = 3,259; African Americans, *n* = 1,745) were used. These subjects were from the cohorts from the following studies: AIDS Clinical Trial Group (ACTG) Study (https://actgnetwork.org), International HIV Controllers Study (3) (http://www.hivcontrollers.org), Multicenter AIDS Cohort Study (MACS) (88) (https://statepi.jhsph.edu/macs/macs.html), Multicenter Hemophilia Cohort Study (MHCS) (89) (https://biolincc.nhlbi.nih.gov/studies/mhcs/), The Study of the Consequences of Protease Inhibitor Era (SCOPE) (90) (https://hiv.ucsf.edu/research/scope.html), and the Swiss HIV Cohort Study (91) (www.shcs.ch).

Viremic controllers were defined as individuals with viral loads of <2,000 copies/ml; noncontrollers were defined as individuals with viral loads of >10,000 copies/ml.

### Tetramer generation and staining.

Peptide-major histocompatibility complex (MHC) tetramers conjugated to fluorophores were generated as previously described, using streptavidin-phycoerythrin (PE) or allophycocyanin (APC) (92). Cytotoxic saporin-conjugated tetramers were produced by the same method using streptavidin-SAP (Advanced Targeting Systems) to tetramerize peptide-MHC monomers according to the published approach (93). Briefly, these modified tetramers are coupled to a toxin, the ribosome-inactivating protein saporin (SAP), which can selectively kill antigen-specific cells of interest and thereby evaluate the contribution of a particular CD8^+^ T-cell specificity to viral inhibition (93–97). The efficiency of tetramerization was confirmed by staining with anti-mouse Ig κ beads (BD Biosciences) with an anti-HLA antibody, followed by tetramer staining. For staining with the fluorescently conjugated tetramers, PBMC or expanded CD8^+^ T cells (0.5 × 10^6^ to 1 × 10^6^ cells per stain) were washed with phosphate-buffered saline (PBS), incubated with relevant tetramers for 20 to 30 min at room temperature in a 96-well U-bottom plate, washed again, further incubated with fluorochrome-conjugated antibodies for 15 min at room temperature, and fixed in 2% formaldehyde solution at 4°C. For staining with SAP-conjugated tetramers, cells were incubated with tetramers for 30 min at room temperature, washed, fixed and permeabilized with the BD Cytofix/Cytoperm kit (BD Biosciences), and then incubated with a secondary anti-SAP antibody (Alexa Fluor 488; Advanced Targeting Systems) as previously published (93). Controls included cells incubated with no tetramer, HLA-mismatched SAP-conjugated tetramers, and free unconjugated SAP. All samples were acquired within 6 h of staining on a MACSQuant Analyzer 10 (Miltenyi Biotec). Negative gates were set up using staining with no tetramer or with HLA-mismatched tetramers. Samples were analyzed in FlowJo version 9.7.6 (Tree Star, Inc.) and hierarchically gated on singlets, lymphocytes, live cells, and CD3^+^ CD4^−^ cells around CD8^+^ tetramer-specific cell populations; in viral inhibition assays, cells were gated on live CD4^+^ green fluorescent protein-positive (GFP^+^) populations.

### Selective depletion of antigen-specific CD8^+^ T cells using cytotoxic tetramers.

Antigen-specific CD8^+^ T cells were selectively depleted using cytotoxic saporin-conjugated tetramers (tet-SAP) as described previously and confirmed in our laboratory (93). First, CD8^+^ T cells within PBMC were expanded using a monoclonal CD3.4 antibody bispecific for CD3 and CD4 (the NIH AIDS Reagent Program) which simultaneously eliminates CD4^+^ T cells and expands CD8^+^ T cells (45–47). Expanded CD8^+^ T cells were cultured in R10 medium (RPMI, 10% fetal calf serum [Sigma], 1% l-glutamine [Sigma], and 1% penicillin-streptomycin [Sigma]), supplemented with 50 U/ml human premium-grade interleukin-2 (IL-2; Miltenyi Biotec) (R10/50) for 10 to 14 days to achieve >90% purity. Expanded CD8^+^ T cells were then treated with tet-SAP (5 to 10 nM) for 2 h at 37°C, washed three times with R10, and cultured in R10/50 for 24 to 48 h before they were used as effector cells in viral inhibition assays (see below). Control treatments included HLA-mismatched tet-SAP, free saporin, or no treatment. Depletion efficiency was confirmed by tetramer staining prior to viral inhibition assay setup. tet-SAP-mediated depletion was prevalidated by depleting antigen-specific cells using PE-conjugated tetramers and anti-PE magnetic beads (StemCell Technologies).

### Generation of polyclonal epitope-specific CD8^+^ T-cell lines.

Epitope-specific CTL lines were generated as previously described (18) with modifications. Briefly, fresh PBMC were peptide pulsed (2 × 10^6^ to 3 × 10^6^ PBMC/peptide at 200-μg/ml final concentration) for 1 h and fed with fresh R10/50 every 2 to 3 days for 14 to 21 days. Specificity was tested by tetramer staining. To remove nonspecific cells, tetramer-positive cells were sorted on a MoFlo XDP cell sorter (Beckman Coulter) and expanded in R10/50 supplemented with monoclonal OKT3 antibody (eBioscience) at 0.1 μg/ml. At the time of the initial setup and every 10 to 14 days afterward, peptide-pulsed irradiated HLA-matched B cells and irradiated feeder PBMC from three HIV-negative donors were added to the sorted cells at a 1:1:1 ratio. The specificity and purity of expanded CD8^+^ T cells were confirmed by tetramer staining immediately before using them as effector cells in viral inhibition assays.

### Generation of CD8^+^ T-cell clones.

Epitope-specific clones were generated as previously described (38). Briefly, PBMC were stained with fluorescently labeled tetramers. Tetramer-specific single cells were sorted on a MoFlo XDP cell sorter (Beckman Coulter) directly into U-bottom 96-well plates (single cell/well) in R10/50 containing monoclonal OKT3 antibody (eBioscience) at 0.1 μg/ml. Twice a week, half of the medium was replaced with fresh R10/50. After 2 to 3 weeks, cells were tested for their specificity by tetramer staining. Epitope-specific clones were transferred to 48-well plates and then to 24-well plates; at the time of transfer and/or every 14 to 21 days, clones were restimulated with monoclonal OKT3, peptide-pulsed irradiated HLA-matched B cells, and irradiated feeder PBMC from three HIV-negative donors.

### Viral inhibition assays.

To evaluate anti-HIV suppressive capacity of *ex vivo* unstimulated CD8^+^ T cells or stimulated epitope-specific CD8^+^ T cells, we modified a previously described viral inhibition assay (98). We used an HIV-permissive T1 cell line untransfected or transfected with the HLA-B*14:02 gene (provided by Otto Yang; this cell line also expresses HLA-A*02, HLA-B*05, and HLA-B*06) (99) as target cells. Effector cells were (i) “zapped” CD8^+^ T cells, from which Env-EL9- or Gag-DA9-specific cells were selectively depleted using tet-SAP as described above; (ii) CTL lines; and (iii) CD8^+^ T-cell clones. For the initial setup, target cells were infected with pretitrated NL4-3–GFP by spinoculation for 1 h, incubated at 37°C for 1 h, repeatedly washed, and further cultured with or without effector cells in duplicate or triplicate. Every 2 to 3 days, cultures were fed and stained to assess live CD4^+^ GFP^+^ cells. Percent GFP^+^ uninfected target cells served as a background, subtracted from all values. HIV-suppressive capacity was calculated at the time of the peak of viral growth as follows (98): suppressive capacity = log_10_(% GFP^+^ infected target cells without effector cells/% GFP^+^ target cells with effector cells).

The viral inhibition assays shown were done using CTL lines and clones that were generated from 3 subjects.

### Antibodies.

Antibodies used were anti-CD3-brilliant violet 421 (UCHT1), anti-CD4-APC (OKT4), anti-CD4-fluorescein isothiocyanate (FITC) (OKT4) and anti-CD8-PE/Cy7 (RPA-T8) (BioLegend), anti-HLA-APC (G46-2.6) (BD Biosciences), LIVE/DEAD fixable near-infrared (IR) marker (Life Technologies), and polyclonal chicken anti-saporin-Alexa Fluor 488 (Advanced Targeting Systems).

### IFN-γ ELISPOT assays.

Freshly isolated or cryopreserved PBMC were screened in IFN-γ ELISPOT assays to quantify CD8^+^ T-cell responses to a set of 410 overlapping 18-mer peptides spanning the HIV proteome (6) and HLA-restricted optimal epitopes listed in the Los Alamos A-list of optimal HIV CTL epitopes (100). ELISPOT assays were performed as previously described (101, 102). Spots were counted using an automated ELISPOT reader (AID ELISPOT v4.0; Autoimmun Diagnostika, Germany). Positive responses had to be at least three times the mean number of spot-forming colonies (SFC) in the four control wells and >50 SFC/million PBMC after background subtraction. HIV peptides were produced by Schafer-N.

### Measurement of functional avidity.

Functional avidity, or antigen sensitivity, was defined as the concentration of an exogenous peptide required to elicit half-maximal cellular response. Functional avidity of CD8^+^ T cells within PBMC was assessed in *ex vivo* IFN-γ ELISPOT assays by incubating 10^5^ PBMC per well with serial peptide dilutions over a range of 7 log_10_ units in triplicate. The peptides used were wild-type Gag-DA9 and Env-EL9. ELISPOT assays were performed as described above.

### Site-directed mutagenesis of NL4-3.

Y301F, K302R, and Y301F/K302R mutations of HIV Gag sequence as well as K588Q and K588R mutations in Env sequence were introduced, respectively, into the HIV subtype B NL4-3 plasmid by using the QuikChange Lightning site-directed mutagenesis kit (Agilent Technologies) along with custom-designed mutagenesis forward and reversed primers. The forward primers are shown as follows (mutated codons shown in bold): 5′-C CTG GCT GTG GAA AGA TAC CTA **CAG** GAT CAA CAG CT-3′ (Env K588Q), 5′-GAC TAT GTA GAC CGA TTC **TTT** AAA ACT CTA AGA GCC GAG-3′ (Gag Y301F), 5′-T AGA GAC TAT GTA GAC CGA TTC TAT **AGA** ACT CTA AGA GCC G-3′ (Gag K302R), and 5′-A GAC TAT GTA GAC CGA TTC **TTT AGA** ACT CTA AGA GCC GAG CAA G-3′ (Gag Y301F/K302R). All mutations were confirmed by sequencing.

### Virus production and replication kinetics.

All plasmids were maxiprepped according to the manufacturer's instructions (HiSpeed plasmid maxikit; Qiagen, Hilden, Germany). To generate mutant viruses, the mutated NL4-3 Gag-Pro amplified purified PCR products with the BstEII (New England BioLabs, Ipswich, MA)-linearized pNL4-3Δgag-protease were transfected into GFP reporter GXR cells via electroporation in a Bio-Rad Gene Pulsar II using 0.4-cm cuvettes at 300 V, 500 μF, and infinite resistance (14). Virus propagation was then monitored by flow cytometry (LSRII; BD Biosciences) to detect GFP-expressing infected cells for 2 weeks in culture with GXR cells. Virus culture supernatants were harvested when 30% of cells were GFP positive. Viruses were aliquoted and stored at −80°C until use. All mutations were confirmed again by extracting viral RNA from the harvested supernatant and sequencing. Nucleotide similarity reached 99.99%. Along with wild type (wt) as positive controls and two negative controls without viruses, NL4-3 mutant viruses were incubated with GXR cells in a 24-well plate for determination of viral titers, as previously described (103). A low multiplicity of infection (MOI) (0.01%) was set as the lowest threshold for determining the amount of virus required for inoculation. The GFP^+^ expression was measured by flow cytometry from days 2 to 7 before it reached the saturated 30 to 40%. The viral replication capacity was defined by the semilog calculation of the mean slope of exponential growth in Excel. This was further calibrated to the normalized value relative to the wild-type NL4-3, respectively. All assays were done at least in triplicate.

### Amplification and sequencing of proviral DNA.

Genomic DNA was extracted from whole-blood QIAamp reagents (Qiagen, United Kingdom), according to the manufacturer's protocol. For the subjects from the SCOPE cohort, whole blood was unavailable and DNA was extracted from cryopreserved PBMC using the QIAamp DNA minikit according to the manufacturer's protocol (Qiagen). HIV Gag and partial Env (containing HLA-B*14-Env-EL9 ^584^ERYLKDQQL^592^ epitope) segments were amplified by nested PCR, as previously described (104), using the following primers: Gag-specific primers 5′-CTCTAGCAGTGGCGCCCGAA-3′ and 5′-TCCTTTCCACATTTCCAACAGCC-3′ for the first round (product size, 1,418 bp; HXB2 coordinates 627 to 2045) and 5′-ACTCGGCTTGCTGAAGTGC-3′ and 5′-CAATTTCTGGCTATGTGCCC-3′ for the second round (product size, 1,307 bp; HXB2 coordinates 696 to 2003) and Env-specific primers 5′-GGAGATATAAGACAAGCACATTG-3′ and 5′-CCCTGTCTTATTCTTCTAGGT-3′ for the first round (product size, 1,579 bp; HXB2 coordinates 7194 to 8773) and 5′-GTGGAGGAGAATTTTTCTATTG-3′ and 5′-CTATCTGTTCCTTCAGCTACTGC-3′ for the second round (product size, 1,349 bp; HXB2 coordinates 7357 to 8707). PCR products were purified using the QIAquick PCR purification kit (Qiagen, United Kingdom) according to the manufacturer's instructions. All sequencing was done using BigDye Terminator v3.1 Ready Reaction mix (Applied Biosystems) as previously described (104) and analyzed using Sequencher v4.8 (Gene Codes Corp.). We generated maximum-likelihood trees of all sequences, using Mega6.06-mac software and FigTree v1.4.2, to exclude the possibility of contamination with laboratory viral strains. HIV subtypes were further confirmed with NCBI (https://www.ncbi.nlm.nih.gov/guide/sequence-analysis/) and REGA (http://dbpartners.stanford.edu:8080/RegaSubtyping/stanford-hiv/typingtool/) HIV genotyping tools.

### Statistical analysis.

Statistical analyses were performed in GraphPad Prism for Mac OSX, 5.0c (GraphPad Software). We used a paired *t* test to compare differences between remaining infected target cells without effector cells and those with effector cells at the peak of viral replication; the Kruskal-Wallis test with Dunn's posttest (for >2-group analysis) or the Mann-Whitney U test (for 2-group analysis) to analyze differences in HIV suppressive capacity; Fisher's exact test to analyze differences in recognition of Env-EL9 versus Gag-DA9 epitopes and in autologous sequences of Env-EL9 versus Gag-DA9 epitope; the Mann-Whitney U test for differences in magnitude and functional avidity of Env-EL9 versus Gag-DA9 CD8^+^ T-cell responses, in frequency of tetramer-specific cells in HLA-B*14:01- versus HLA-B*14:02-positive subjects, and in viral load and CD4^+^ T-cell counts; Spearman correlation to analyze the correlation between response magnitude and functional avidity; and analysis of variance (ANOVA) with Dunnett's multiple-comparison test for differences in viral replicative capacity of different viral constructs. Functional avidity (EC_50_) was calculated in Prism using a dose-response function.

To analyze associations between HLA class I expression and HIV immune control, SAS 9.2 (SAS Institute) was used. Genotype frequencies on individual HLA-B alleles were computed using PROC FREQ. To calculate OR and 95% confidence interval (CI) for viremic controllers versus noncontrollers with adjustment by HLA-B*27 and HLA-B*57, PROC LOGISTIC was used; an OR of <1 indicates protection. Presence versus absence of all individual HLA-B alleles that have a frequency equal to or greater than HLA-B*14:01 was included in the models with stepwise selection.

### Accession number(s).

Sequences were deposited in GenBank under accession numbers MF445302 to MF445379.
